# Neutrophil Gelatinase-Associated Lipocalin as an Early Sign of Diabetic Kidney Injury in Children

**DOI:** 10.4274/jcrpe.2002

**Published:** 2015-12-03

**Authors:** Zeynep Yürük Yıldırım, Ahmet Nayır, Alev Yılmaz, Asuman Gedikbaşı, Rüveyde Bundak

**Affiliations:** 1 İstanbul University İstanbul Faculty of Medicine, Department of Pediatric Nephrology, İstanbul, Turkey; 2 Dr. Sadi Konuk Training and Research Hospital, Clinic of Biochemistry, İstanbul, Turkey; 3 İstanbul University Istanbul Faculty of Medicine, Department of Pediatric Endocrinology, İstanbul, Turkey

**Keywords:** Diabetic nephropathy, Microalbuminuria, neutrophil gelatinase-associated lipocalin, diabetes mellitus

## Abstract

**Objective:**

There is some evidence indicating that histopathological changes in type 1 diabetes mellitus (T1DM) emerge before onset of microalbuminuria. The aim of our study was to determine whether urine neutrophil gelatinase-associated lipocalin (NGAL) levels can be considered as an early sign of diabetic kidney injury.

**Methods:**

Urine NGAL (uNGAL) levels and urinary NGAL/creatinine ratio (uNGAL/Cr) were assessed in 76 patients with T1DM and compared with the findings of 35 healthy individuals. The relationship of uNGAL levels with diabetes duration, body mass index (BMI), serum lipids, HbA1c, and microalbuminuria was also evaluated.

**Results:**

Mean uNGAL (100.16±108.28 ng/mL) and uNGAL/Cr (118.93-117.97 ng/mg) levels in both microalbuminuric and non-microalbuminuric diabetic patients were found to be higher than those in the control group (uNGAL: 21.46±18.59 ng/mL and uNGAL/Cr: 32.1±51.48 ng/mg) (p=0.0001).

**Conclusion:**

Urine NGAL level increases in the very early phase of T1DM before microalbuminuria develops. The patients with T1DM should be considered to have diabetic kidney injury from the time of diagnosis on and preventive interventions need to be initiated at an early stage to preclude the progression to end-stage renal disease.

**WHAT IS ALREADY KNOWN ON THIS TOPIC?**

SOnly a few studies have evaluated urine neutrophil gelatinaseassociated lipocalin levels in diabetic adults and children previously.

**WHAT THIS STUDY ADDS?**

The main finding of our study was that urine neutrophil gelatinase-associated lipocalin and urine neutrophil gelatinaseassociated lipocalin/creatinine ratio were significantly higher in diabetic patients than in control subjects. Interestingly, urine neutrophil gelatinase-associated lipocalin levels were higher in non-microalbuminuric patients than in controls in our study. Our study group was the largest pediatric patient group so far and the present study also evaluated a possible relationship between urine neutrophil gelatinase-associated lipocalin levels and factors affecting the progression of kidney injury in type 1 diabetes mellitus, unlike the other studies.

## INTRODUCTION

Type 1 diabetes mellitus (T1DM), the most common endocrine disorder of childhood, leads to various macro- and microvascular complications (1). Diabetic nephropathy (DN) is one of the major microvascular complications of T1DM and results in end-stage renal disease (ESRD) (2). Nowadays, the earliest sign of DN is considered to be microalbuminuria (3). However, it has been known that some histopathological changes related to DN emerge before the onset of microalbuminuria (4). Therefore, noninvasive methods to diagnose DN in earlier stages have been investigated in some studies (5,6,7). These studies mostly focused on neutrophil gelatinase-associated lipocalin (NGAL) which is the most promising biomarker for early detection of acute kidney injury (AKI) (8,9,10). NGAL, a member of the lipocalin protein superfamily is released from the renal tubular cells in response to various acute and chronic insults to the kidney such as contrast nephropathy, IgA nephropathy, lupus nephritis (11,12,13,14). Renal functional deterioration has been demonstrated to be associated with tubulointerstitial injury as well as glomerular changes in DN (15). Hence, it has been considered that NGAL as a tubular marker may predict diabetic renal damage (6).

Although DN rarely becomes manifest in childhood, the changes that lead to deterioration of kidney functions are known to begin in childhood. Therefore, we aimed to assess whether urinary NGAL (uNGAL) levels can predict the early phases of diabetic kidney injury in childhood. We also evaluated a possible relationship between uNGAL levels and the factors affecting progression of kidney injury in T1DM.

## METHODS

Seventy-six consecutive T1DM patients who applied to the outpatient clinic of the Pediatric Endocrinology Department of the İstanbul University İstanbul Medical Faculty for routine follow-up between July and October 2011 and 35 healthy individuals were enrolled in the study. All T1DM patients were on intensive insulin treatment or insulin pump treatment. After informed consent was obtained, the patients underwent a standard physical examination and blood samples were drawn for biochemical analysis. The body mass index (BMI) was evaluated based on percentile curves of Turkish children (16). Estimated glomerular filtration rate (eGFR) using the Schwartz et al (17) formula was normal in all patients. A complete urinalysis and a urine culture were performed and urinary tract infection was excluded in all patients. Patients were considered microalbuminuric if they had a urine microalbumin/creatinine ratio (uMA/Cr) greater than 30 mg/g Cr in at least two out of three urine samples during outpatient follow-up (18).

Urinalysis including leukocyte esterase reaction, nitrite test, and microscopic analysis of the urine was performed by Iris IQ 200 full automatic urine analyzer.

First morning urine specimens were obtained to measure urine creatinine, uMA, and uNGAL. Immediately after being collected, urine samples were centrifuged at 15000 g, 4 °C for 15 minutes, the supernatant was removed, and the samples were stored at -80 °C, to be later analyzed all at the same time. The NGAL/Lipocalin-2 ELISA (Enzyme-Linked Immunosorbent Assay) kit (BioVendor, RD191102200R) was used to measure uNGAL according to protocol, with uNGAL levels expressed in ng/mL and urinary NGAL/creatinine ratio (uNGAL/Cr) values expressed in ng/mg. The Abbott Architect c16000 analyzer was used to measure uCr and uMA, with uMA expressed in mg/L and uMA/Cr expressed in mg/g.

Approval for this study was obtained from the Ethics Committee of the İstanbul University İstanbul Medical Faculty.

### Statistical Analysis

All statistical analyses were performed using the Number Cruncher Statistical System 2007 Statistical Software (Utah, USA). In addition to descriptive statistical methods (mean, standard deviation, median and interquartile range), non-parametric methods were used for non-normally distributed variables (the Kruskal-Wallis test for comparisons of groups, Dunn’s multiple comparison test for comparisons of subgroups, the Mann-Whitney U test for comparisons of two groups). Parametric methods were used for normally distributed variables (one-way analysis of variance for comparisons between groups, Tukey’s multiple comparison test for comparisons of subgroups, independent t-test for comparisons between two groups, the chi-square test for comparisons of qualitative variables). In order to determine cut-off values for uNGAL and uNGAL/Cr and their significance in differential diagnosis, area under the receiver operating characteristic (ROC) curve, sensitivity, specificity, positive and negative predictive values, as well as positive and negative likelihood ratios were calculated. Results were evaluated with the level of significance set at p=0.05 and a 95% confidence interval.

## RESULTS

The patient group consisted of 76 children (36 male and 40 female) and the control group of 35 children (18 male and 17 female). The mean ages of the patient and control groups were 12.43±3.87 years and 11.14±3.77 years, respectively. All patients had normal blood pressure and eGFR; the mean eGFR was 155±30 mL/min/1.73 m2. Mean BMI values were 19.11±3.92 (9.87-32.12) in the patient group and 17.84±3.4 (11.97-27.7) in the control group. There were no significant differences between the patient and control groups in terms of gender, age, and BMI (p>0.05).

**Figure 1:**
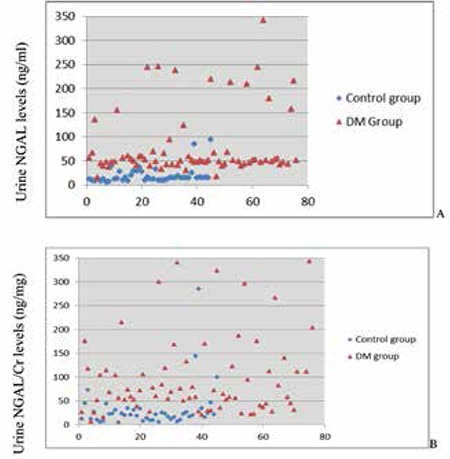
The distribution of urine neutrophil gelatinase-associated lipocalin (uNGAL) levels in patient and control groups A: for uNGAL (ng/ml), B: for urine NGAL/creatinine ratio (uNGAL/Cr) (ng/mg)

**Figure 2:**
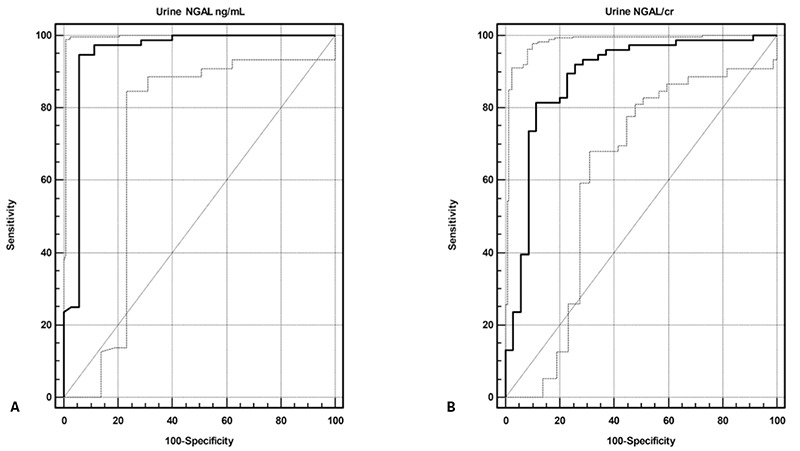
Receiver operating curve to detect diabetic kidney injury A: for urine neutrophil gelatinase-associated lipocalin (NGAL) (area under the curve (AUC)=0.948), B: for urine neutrophil gelatinase-associated lipocalin/creatinine ratio (uNGAL/Cr) (AUC=0.886)

Mean uNGAL levels were significantly higher in the patient group than in the controls (100.16±108.28 ng/mL vs 21.46±18.59 ng/mL; p=0.0001) (Figure 1A). ROC analysis showed that an uNGAL level of 36.3 ng/mL was the optimal cut-off value for predicting diabetic kidney injury. With this cut-off value, sensitivity was 94.74%, specificity was 94.29%, the positive predictive value was 97.3%, and the negative predictive value was 89.2%. When uNGAL level is above this cut-off value, the possibility of diabetic kidney injury increases 16-fold (positive likelihood ratio of 16.58; negative likelihood ratio of 0.056). Area under the curve (AUC) was 0.948 (Figure 2A).

Mean uNGAL/Cr values were also significantly higher in the patient group than in the control group (118.93±117.97 ng/mg vs. 32.1±51.48 ng/mg; p=0.0001) (Figure 1B). ROC analysis revealed that the optimal cut-off value to predict diabetic kidney injury was 34.88 ng/mg for uNGAL/Cr. For this cut-off value, sensitivity was 81.58%, specificity was 88.57%, positive predictive value was 93.9%, and negative predictive value was 68.9%. Above this cut-off value, the possibility of diabetic kidney injury increased 7 fold (positive likelihood ratio of 7.14; negative likelihood ratio of 0.21). AUC was 0.886 (Figure 2B).

Eleven (14.5%) of the patients had microalbuminuria. Both uNGAL and uNGAL/Cr were higher in microalbuminuric and non-microalbuminuric patients than in controls (p<0.05). Although the difference was not statistically significant, the patients with microalbuminuria had higher uNGAL levels than those without microalbuminuria (145.95±138.29 ng/mL vs. 92.41±101.63 ng/mL, p=0.270). A positive correlation was found between uNGAL and uMA levels (r=0.344, p=0.002) (Table 1).

The mean diabetes duration after the first diagnosis of T1DM was 44.4±35.4 months (0.8-212 months). According to the diabetes duration, the patients were divided into 3 subgroups: those with a diabetes duration of 0-2 years (n=31), 2-5 years (n=23), and greater than 5 years (n=22). Although there were no significant differences between these three subgroups in terms of uNGAL and uNGAL/Cr (p>0.05), uNGAL levels in the patients who had a T1DM duration of 2-5 years and >5 years were 26% higher than in those of the 0-2 years group (Table 2). When each subgroup was compared with the control group, uNGAL and uNGAL/Cr were found to be higher in each subgroup than in the control group (p=0.0001).

Based on HbA1c levels, patients were divided into 3 subgroups as those with good glycemic control (HbA1c: 6.5-7.5%; n=20), moderate glycemic control (HbA1c: 7.5-9%; n=31), and poor glycemic control (HbA1c >9%; n=25). There were no significant differences between these groups regarding uNGAL (p=0.423), uNGAL/Cr (p=0.371), and uMA (p=0.112) (Table 3).

A positive correlation was noted between uNGAL and BMI (r=0.286, p=0.012). There was no correlation of uNGAL levels with cholesterol, triglycerides, high-density lipoprotein (HDL), low-density lipoprotein (LDL), very low density lipoprotein (VLDL), and HbA1c levels (p>0.05). Also, uNGAL/uCr did not correlate with uMA, uMA/uCr, cholesterol, HDL, LDL, VLDL, HbA1c levels, and BMI (p>0.05).

## DISCUSSION

It is well known that DN progresses with time elapsed following the initial diagnosis of diabetes. The early changes in DN such as hyperfiltration, widening of the glomerular basement membrane, and microalbuminuria are followed by increased accumulation of extracellular matrix and impaired kidney functions (19). The current approach to DN in diabetic patients is to initiate the renin-angiotensin-system (RAS) inhibition once microalbuminuria emerges. Microalbuminuria is usually detected 5-10 years after the diagnosis of T1DM (20,21,22). However, diabetic kidney injury begins before onset of microalbuminuria. Therefore, some measurements to prevent DN are needed to be initiated before microalbuminuria emerges. Moreover, the effect of these preventive measurements will be seen 5-10 years after they were initiated (19,23). Fioretto et al (24) performed successful pancreas transplantation in 8 patients with biopsy proven DN, and these patients remained insulin-independent for 10 years following the transplantation. According to the results of this study, microalbuminuria improved after 5 years and histopathologic findings of DN ameliorated after 10 years (24). Hence, the early recognition of diabetic kidney injury before the microalbuminuric phase is essential to prevent ESRD related to DN. As a tubular biomarker, uNGAL might be a good candidate for detection of diabetic kidney injury prior to the microalbuminuric phase because it has been shown that tubular impairment has an important role in the pathogenesis and progression of DN.

The main result of our study is that uNGAL and uNGAL/Cr were significantly higher in diabetic patients than in the control subjects. Only a few studies have evaluated uNGAL levels in diabetic adults and children previously (5,6,25,26). Bolignano et al (5) found higher uNGAL levels and fractional excretion rates of NGAL in adults with type 2 DM than in controls. uNGAL/Cr was reported to be higher in patients with T1DM than in the control group in adults by Nielsen et al (6) and in normoalbuminuric adolescents by Demir et al (26). Zachwieja et al (25) also reported higher uNGAL levels in non-microalbuminuric children with T1DM than in controls. At this point, the most important question is whether elevated uNGAL levels or uNGAL/Cr indicate diabetic kidney injury before microalbuminuria occurs. Our study confirms previous reports which documented that uNGAL and uNGAL/Cr were higher in non-microalbuminuric patients than in healthy controls (5,6,25,26). This finding indicates that the elevation of uNGAL and uNGAL/Cr in diabetic kidney injury occurs before microalbuminuria emerges. Theoretically, it may be expected that uNGAL/Cr is also much higher in microalbuminuric patients than in non-microalbuminuric children. However, we could not demonstrate this difference in our study probably due to the limited sample size of the microalbuminuric group.

The second question is when the diabetic kidney injury starts in T1DM. To answer this question, we compared uNGAL levels of the patients with different durations of diabetes by dividing them into three groups arbitrarily according to duration of diabetes as 0-2 years, 2-5 years, and >5 years. uNGAL levels and uNGAL/Cr were significantly higher in each of these groups than in healthy controls. We could not demonstrate significant differences among these three subgroups in terms of uNGAL and uNGAL/Cr. However, although the difference did not reach statistical significance, uNGAL levels in patients with a diabetes duration of 2-5 years and >5 years were found to be higher than in patients with a duration of 0-2 years. This result is in accordance with the only previously reported study which showed that uNGAL increased 15% a year in adults with T1DM (7). In our study, we found higher uNGAL levels and uNGAL/Cr even in diabetic patients with 0-2 years duration compared to healthy controls, a finding suggesting that diabetic kidney injury possibly begins in a very early phase of T1DM.

Another question is whether uNGAL or uNGAL/Cr is related to predictors of progression in DN. Various predictor factors have been identified in DN such as high HbA1c levels, elevated serum lipids, high blood pressure, and increased body weight. Similar to previous reports, uNGAL and uNGAL/Cr were not related to any of these factors in our study group. The last question is whether uNGAL or uNGAL/Cr can be used as a marker to predict diabetic kidney injury in practice. Our results suggest that uNGAL and uNGAL/Cr may be a good indicator for diabetic kidney injury in pre-determined cut-off levels with high sensitivity and specificity. However, our study has some limitations. One of them was that the number of patients with microalbuminuria was small and this may be the reason why we could not find any difference between microalbuminuric and normoalbuminuric patients in terms of uNGAL/Cr. Studies in larger patient groups are needed to confirm these results, and adolescents may be evaluated as a different group in these studies because the control of diabetes may be problematic in adolescents. The other limitation was that analysis of uNGAL and uNGAL/Cr was based on one sample. The monitoring of NGAL levels and uNGAL/Cr at regular intervals in order to evaluate their dynamics over the course of years and their correlation to other parameters in T1DM would possibly give important clues as to the clinical use of these parameters.

In conclusion, uNGAL level which is a specific biomarker for AKI increases in a chronic progressive kidney disease, diabetic kidney injury. This increase occurs in very early phase of the disease, before the onset of microalbuminuria. The patients with T1DM should be considered as diabetic kidney injury from the time of diagnosis on and preventive interventions are needed to preclude the progression to ESRD.

## Figures and Tables

**Table 1:**
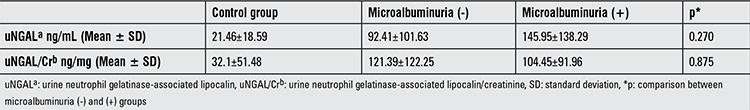
Urine neutrophil gelatinase-associated lipocalin and urine neutrophil gelatinase-associated lipocalin/creatinine in patients with and without microalbuminuria and in healthy controls

**Table 2:**
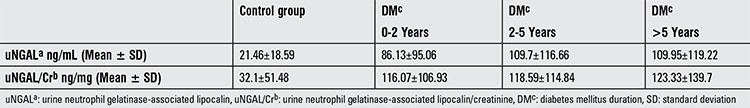
The relationships between urine neutrophil gelatinase-associated lipocalin and urine neutrophil gelatinase-associated lipocalin/creatinine and diabetes duration

**Table 3:**
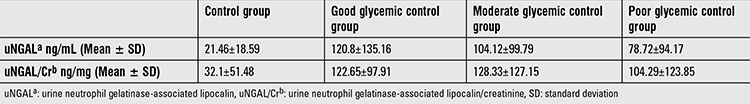
The relationships between urine neutrophil gelatinase-associated lipocalin and urine neutrophil gelatinase-associated lipocalin/creatinine and glycemic control
